# Study on the Preparation of Plasma-Modified Fly Ash Catalyst and Its De–NO_X_ Mechanism

**DOI:** 10.3390/ma11061047

**Published:** 2018-06-20

**Authors:** Lei Zhang, Xin Wen, Lei Zhang, Xiangling Sha, Yusu Wang, Jihao Chen, Min Luo, Yonghui Li

**Affiliations:** 1 School of Geology and Environment, Xi’an University of Science and Technology, Xi’an 710054, China; wenxin_xust@126.com (X.W.); isee_wys@163.com (Y.W.); chenchenjijihaohao@126.com (J.C.); lm876098550@126.com (M.L.); 2 China National Heavy Machinery Research Institute Co., Ltd., Xi’an 710032, China; zlcnhmri@126.com (L.Z.); xzslyh@126.com (Y.L.); 3 Shandong Baichuan Tongchuang Energy Co., Ltd., Jinan 250101, China; shaxiangling@126.com

**Keywords:** denitration, fly ash catalyst, plasma modification

## Abstract

Fly ash and bentonite were mixed in a certain proportion as raw materials to prepare a denitration catalyst. In previous studies, it has been concluded that fly ash-type catalysts can provide significant catalytic activity for denitrification after being modified with oxygen. In this study, the effect of plasma conditions on the denitration performance of the catalyst was investigated from the aspects of plasma modification power, modification time, and the flow rate of the gas. Boehm titration and infrared analysis systems were used to characterize the performance of the catalyst. The experimental results show that the optimal modification power is 60 W, the optimal modification time is 20 min, and the optimal gas flow rate is 40 mL/min.

## 1. Introduction

In recent years, low-temperature plasma technology has gotten many industrial applications, due to its ability to activate and convert reactive molecules at room temperature, and it is particularly favored in flue gas denitrification. Low-temperature plasma technology can simultaneously remove a variety of pollutants during the denitration reaction, and can effectively reduce the secondary pollution caused to the environment. Therefore, it is an ideal flue gas treatment technology for the purpose of thoroughly purifying the coal-fired flue gas [1,2,3]. However, there are also problems with the technology, such as the low efficiency of denitration, wide distribution of products, and poor selectivity of target products. In addition, the most important feature of the catalytic reaction is the use of the catalyst to speed up the main reaction rate, suppress the side reactions, and increase the selectivity of the target product—that is, accelerate the main reaction during the catalytic reaction and suppress the side reactions. Therefore, the synergy between low temperature plasma technology and catalytic technology can achieve the effect of complementing each other and further increase the efficiency of denitration [4,5].

Fly ash is a kind of special powder with a hollow structure and solid structure, a porous structure and regular structure, organic matter and inorganic matter, all mixed with one another [6,7]. Utilizing the good reactivity, adsorption, and catalytic performance of the special structure of fly ash to treat nitrogen oxides can not only reduce the impact of excess fly ash accumulation on the environment, but also improve global environmental pollution. By using low-temperature plasma to modify fly ash, the feasibility of applying plasma-modified fly ash in flue gas denitrification is explored, and solutions are provided for the safe disposal of fly ash in thermal power plants and their comprehensive utilization of the material.

At present, the main catalyst modification methods include acid modification, alkali modification, and surfactant modification [8]. The plasma can also be used to modify the surface of fly ash, in which case the principle is to apply high voltage first to the active particles in the plasma, such as electrons, ions, radicals, photons, and excited molecules, then generate a strong electric field around each fly ash particle to stimulate more high-energy electrons and free active particles, such as O, OH, and HO_2_. In addition, because the modified fly ash has a large specific surface area, it is more conducive to the enrichment of substances, such as pollutants and free radicals on the fly ash surface. This not only improves the energy efficiency of the fly ash, but also facilitates the removal of pollutants. At the same time, using the modified fly ash as a catalyst carrier is expected to improve the surface properties and surface energy of the catalyst [9,10,11]. The increase of the surface energy contributes to the introduction of reactive groups in the subsequent catalyst, and also improves the uneven distribution of the active components caused by the calcination stage during the preparation of the catalyst. Therefore, using plasma to modify the surface of fly ash can improve its denitrification effect, so that the fly ash type catalyst has a broader prospect in the field of denitrification. Besides, it has been concluded in previous studies that the denitrification effect of the catalyst is significantly better when oxygen is used to modify the fly ash rather than other gases.

Therefore, oxygen modification was used in this paper to study the effect of different plasma modification conditions on the performance of the catalyst. The catalyst performance was characterized by Boehm titration and infrared analysis.

## 2. Materials and Methods

### 2.1. Materials

The experimental raw material for this study was fly ash from the combustion of circulating fluidized bed (CFB) in the Yulin Thermal Power Plant, and the elemental analysis and the ICP analysis of the raw materials was as shown in Table 1 and Table 2 [12]:

### 2.2. The Preparation of Catalysts and Activity Evaluation 

**Figure 1:**
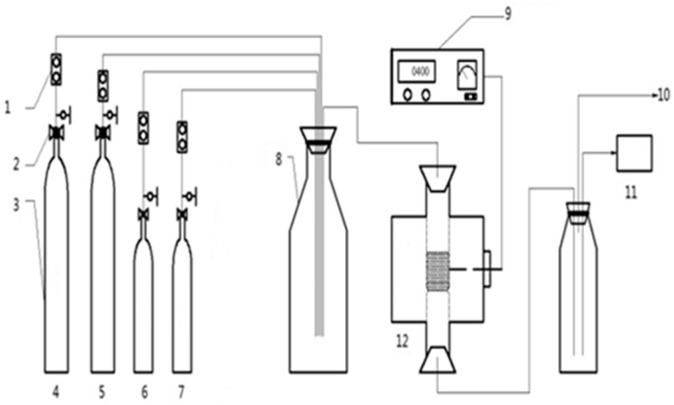
Diagram of the experiment process. (1) Flow meter, (2) pressure relief valve, (3) high-pressure gas cylinder, (4) oxygen, (5) nitrogen, (6) nitric oxide, (7) hydrocarbon gas, (8) mixed gas cylinders, (9) plasma power supply system, (10) fume emission, (11) flue gas analyzer, and (12) plasma reactor.

The fly ash and bentonite were mixed in a 2:1 ratio, and some water was added to mix them evenly. The mixture was then dried and cut into 2 mm sized particles, which we named fly ash catalyst. In each experiment, 1 g of the catalyst was used. The activity evaluation apparatus of the catalyst is shown in Figure 1.

The catalysts were placed in the plasma rector for denitration with simulated flue gas. The simulated flue gas flow *G* was at 1 L/min; the concentration of NO was 450 ppm, the oxygen content was 6%, and the rest was nitrogen. The NO concentration in the tail gas was analyzed and evaluated by the flue gas detector (Testo340, Testo, Germany). 

## 3. Results

### 3.1. The Modification of the Fly Ash Catalyst with Different Gases

**Figure 2:**
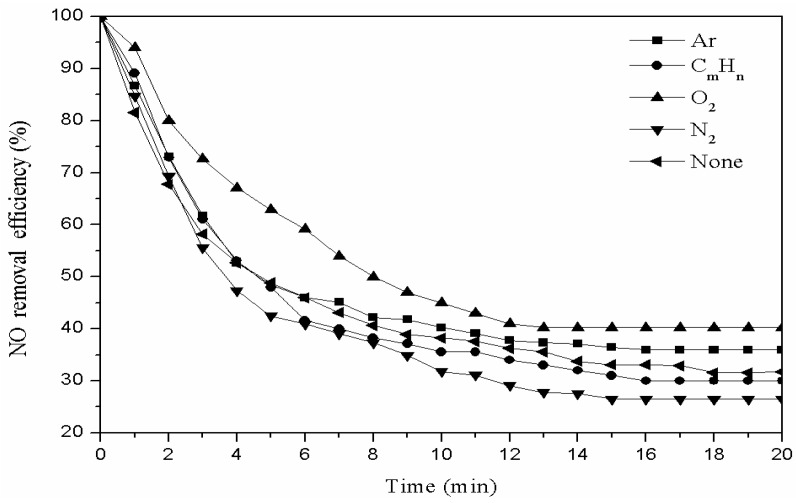
Effect of different modification gases on denitration efficiency.

A total of 3 g of fly ash catalyst was weighed and placed into the plasma reactor. The catalyst was then modified with different gases (nitrogen, oxygen, argon, and hydrocarbon). The power of the plasma reactor was set to 60 W, gas flow rate to 40 mL/min, and the modification time to 20 min. One gram of the modified catalyst was placed into the plasma reactor for the denitration reaction, in order to choose the best modified gas. The denitration efficiency with different modify gases is shown in Figure 2.

The composition of the hydrocarbon gas in this study was ethane (0.998%), acetylene (0.997%), propane (0.5%), propylene (0.2%), methyl (0.199%), acetylene (0.198%), butane (0.199%), butene-1 (0.199%), ethyl acetylene (0.2%), and nitrogen (96.31%).

As Figure 2 shows, the fly ash catalyst that was modified with different gases had different degrees of denitration efficiency. Oxygen has the best effect, and the hydrocarbon gases followed. When the fly ash catalyst was modified with hydrocarbon gas, part of hydrocarbon gas was adsorbed to the catalyst pores and occupied the active sites. The denitration mechanism is as follows:

(1) $$\;C_{x}H_{y}+\mathrm{NO}+O_{2}\to {\mathrm{CO}}_{2}+N_{2}+H_{2}O$$

(2) $$\;C_{x}H_{y}+2O_{2}\to {\mathrm{CO}}_{2}+H_{2}O$$

However, when the fly ash catalyst was modified with Ar and N_2_, the denitration rates were lower than the raw catalyst. Therefore, oxygen was selected as a modified gas [12].

### 3.2. Parameters When Using Oxygen for Modification

#### 3.2.1. Oxygen Modification Time

**Figure 3:**
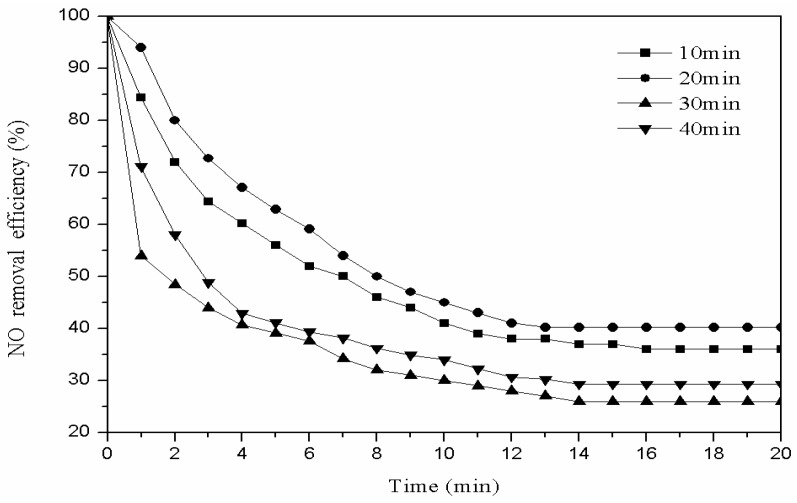
Effect of different modification times on denitration efficiency.

We weighed 3 g of the catalyst modified with oxygen and placed it into the plasma reactor. The plasma reaction power was set to 60 W, the gas flow rate to 40 mL/min, and the modification times to 10 min, 20 min, 30 min, and 40 min. We used 1 g of the modified catalyst for the denitration reaction with low-temperature plasma technology, in order to choose the best modification time. The denitration efficiency with the different modification times is shown in Figure 3.

As Figure 3 shows, the denitration rate increases at first, and then decreases with the modification time increase. When the oxygen modification time was 10 min, less energy was injected into the system and fewer active particles were generated, so the denitration rate was low.

With the modification time increase, more high-energy oxygen particles were ionized by the system, and more energy was injected into the system. The energy etched the organic substance on the catalyst and improved its chemical properties, so the denitration rate increased. However, when the modification time was too long, more high-energy particles impinged on the catalyst surface. Then, the formed active sites were damaged and the pore structure of the catalyst was ruined, thus the catalyst denitration effect was reduced [13]. Therefore, the catalyst denitration effect is best when the oxygen modification time is 20 min.

#### 3.2.2. Plasma Power of the Modification

**Figure 4:**
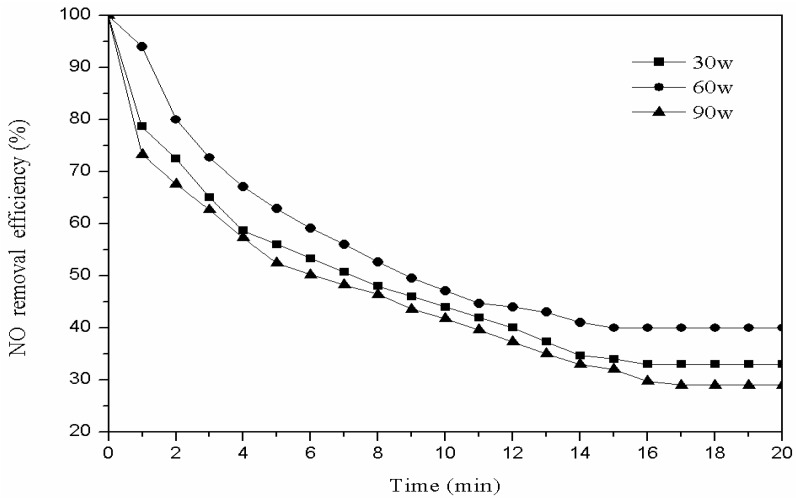
Effect of different plasma power levels on denitration efficiency.

We weighed 3 g of the catalyst modified with oxygen and placed it into the plasma reactor. The oxygen flow rate was set to 40 mL/min, the modification time to 20 min, and the modification plasma power to 30 W, 60 W, and 90 W. A total of 1 g of the modified catalyst was used for the denitration reaction with low-temperature plasma technology, to choose the best modification plasma power level. The denitration efficiency with different modification plasma power levels is shown in Figure 4.

Figure 4 shows that the plasma power of the modification has a great influence on the denitrification efficiency. With the plasma power increase, the denitration rate increased at first, and then decreased. When the plasma power was 30 W, the plasma discharge intensity was insufficient and there were fewer high energy oxygen particles in the system, so no obvious etching effects occurred on the surface of the catalyst; thus, the modification effect was poor. When the modification power was 90 W, the plasma discharged intensely and a clearly violet flame could be observed. At this time, the surface of the catalyst was constantly hit by the high-energy oxygen electron flow. The performance of the functional and pore structures were destroyed and the active sites were inactivated, so the denitration efficiency was reduced [14,15]. Therefore, when the plasma power is 60 W, the catalyst has the best denitration effect.

#### 3.2.3. Modification Gas Flow

**Figure 5:**
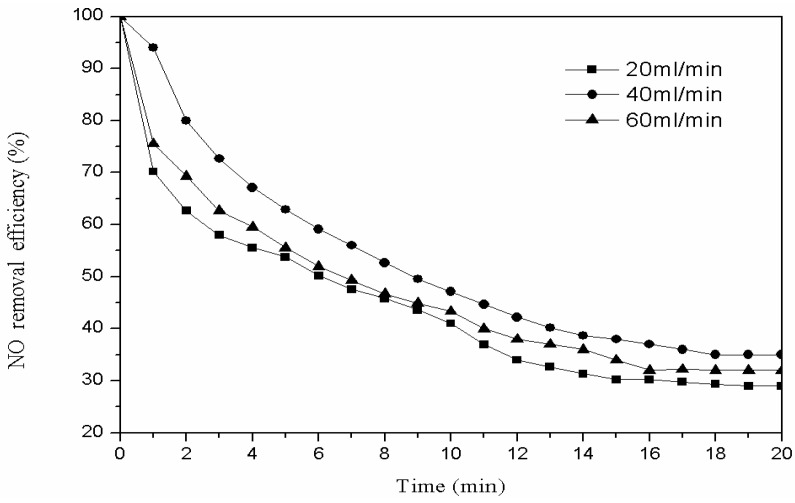
The denitration efficiency with different modified gas flows.

We weighed 3 g of the catalyst modified with oxygen and placed it into the plasma reactor. The power of plasma reactor was set to 60 W, the modification time to 20 min, and oxygen flow rate to 20 mL/min, 40 mL/min, and 60 mL/min. One gram of modified catalyst was used for the denitration reaction with low-temperature plasma technology, to choose the best modification gas flow. The denitration efficiency with different modification gas flows is shown in Figure 5.

It can be seen from Figure 5 that the denitration effect first increased and then decreased with the modification gas flow increase. When the modification gas flow was 20 mL/min, the number of high-energy oxygen particles produced by the plasma discharge was small, and carried away by the oxygen flow before the particles reacted with the surface of catalyst fully. When the modification gas flow was 60 mL/min, a large amount of high-energy oxygen particles could be produced in the plasma reactor. However, the content of the ozone also increased significantly and was absorbed in the catalyst pore structure. In the denitration experiment, more ozone in the pore structure would oxidize NO to NO_2_, and NO_2_ would occupy the active site, which decreases the denitration effect.

Therefore, when the modification flow rate is 40 mL/min, when the plasma discharge intensity is moderate and the active sites are more numerous, the denitration efficiency is the best.

### 3.3. Characterization

In this paper, the fly ash catalyst was modified by plasma reactor. In the modification process, the plasma discharge produces high-energy oxygen particles; high-energy oxygen particles bombard the fly ash catalyst constantly, so that the functional groups have been changed on the catalyst surface. Therefore, the oxygen functional groups on the catalyst surface were characterized by Boehm titration and FTIR characterization (VERTEX 70, Bruker, Billerica, MA, USA).

#### 3.3.1. Characterization of Catalysts Prepared by Different Modification Time 

**Figure 6:**
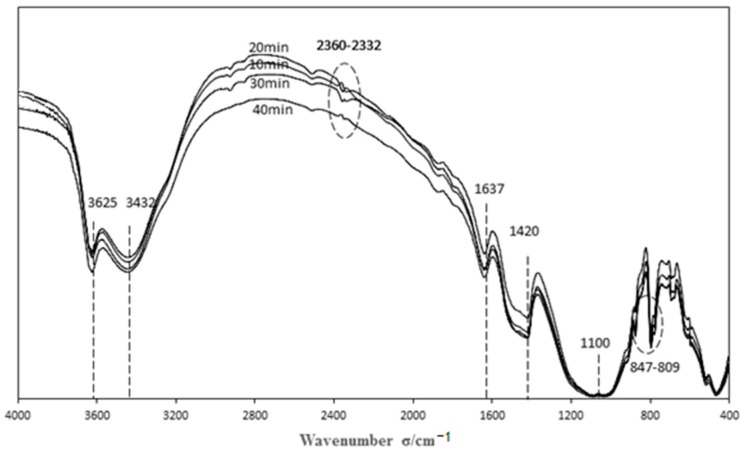
The FTIR spectra with different modification times.

In the oxygen atmosphere, the Boehm titration data for the catalysts with different modification times are shown in Table 3, and the FTIR spectrum is shown in Figure 6.

As the Table 3 shows, there are four types of functional groups on the catalyst surface: acid functional groups, basic functional groups, phenolic hydroxyl groups, and carboxyl groups. Different modification times have different effects on the number of different functional groups, especially acidic and basic functional groups. It can be seen from Table 3 that the number of basic functional groups is very small when the number of acidic functional groups is large, and the number of acidic functional groups is very small when the number of basic functional groups is large. When the modification time is 20 min, the number of acidic functional groups is very small, and the number of basic functional groups is at its largest.

Figure 6 shows the FTIR spectra of different modification times. It can be seen from Figure 6 that the modification time has little effect on the functional groups of the fly ash catalyst. Combined with Table 1, a great gap exists between the number of acidic functional groups and basic functional groups. Since the point at wavenumber 1637 (Figure 6) shows the characteristic peaks of C=O and N=O, C=O belongs to both the carboxyl and the carbonyl group; therefore, it belongs to the acidic functional group. In contrast, N=O belongs to nitrous acid, which is the basic functional group; however, both functional groups play a positive role in the denitration process [16,17]. Combined with Figure 3, it can be seen that when the oxygen modification time is 20 min, the denitration effect of the catalyst was significantly the best. The peak types of C≡C and C≡N at 2360~2332 changed greatly. This is because when oxygen modification time is shorter, the carboxylic acid functional groups were formed on the catalyst surface, and they can play a positive role in the denitration effect [18]; however, when the oxygen modification time is longer, the functional groups on the catalyst surface are bombarded strongly by high-energy oxygen particles, and it would ionize the C≡N, which is difficult to ionize. In the meantime, since the functional groups of C=O and N=O on the catalyst surface were broken, it may form a more stable macromolecular structure and lose part of the active site, so that the denitration rate decreased. Combined with the Figure 3, it can be seen that the denitration effect is best when the catalyst modification time is 20 min followed by 10 min; this was consistent with previous speculation [19]. Therefore, the denitration effect of the basic functional group N=O on the catalyst surface is greater than that of the acid functional group C=O.

#### 3.3.2. Characterization of Catalysts Prepared with Different Modification Power Levels

**Figure 7:**
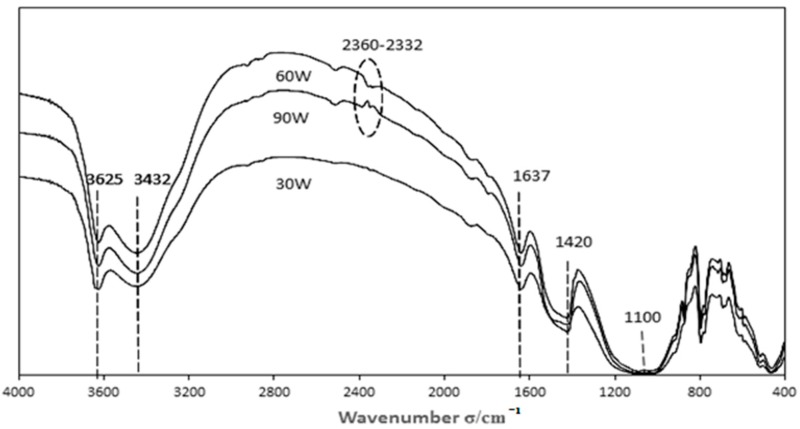
FTIR spectra with different plasma-modified power levels.

The modification power of the plasma has a great influence on the oxygen functional groups of the catalyst surface. The Boehm titration data is shown in Table 4 and the FTIR spectrum is shown in Figure 7.

As can be seen from Table 4, the modification power has little effect on the functional groups on the catalyst surface. The number of acid functional groups and basic functional groups on the catalyst surface are greater when the modification power is 60 W.

As can be seen from Figure 7, when the plasma power is 60 W, the number of the function group O–H at waves 3636 and 3432, the C=O and N=O at 1637, and the –O– at 1100 were all significantly increased This indicates that the functional groups on the catalyst surface after oxygen modification, such as carboxyl, hydroxyl, and carbonyl groups, have a positive effect on denitration [20,21]. In addition, the functional groups of C≡C and C≡N at 2360~2332 on the catalyst surface have changed greatly. The peak type almost disappears when the modification power is 30 W, and disappears completely when the power is at 90 W. Besides, we can tell from Figure 4 that the effect of power on denitration rate is ranked as 60 W > 30 W > 90 W. When the modification power was 30 W, the applied voltage was small and just reached the breakdown voltage of the plasma discharge—that is, the discharge was insufficient. When the modification power was 90 W, the effective discharge time was too long, and its intensity was too great, so the effective functional group which had just been formed was destroyed; even the original active site of the fly ash catalyst was also destroyed, causing the denitration rate to decrease rapidly.

#### 3.3.3. Characterization of Catalysts Prepared by Different Gas Flows

**Figure 8:**
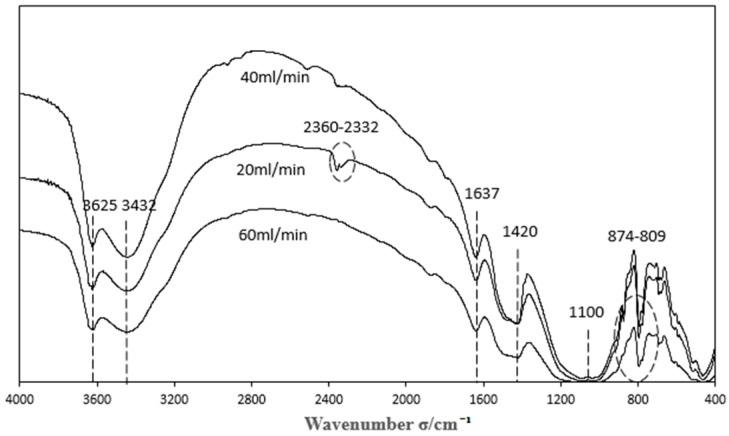
FTIR spectra with different oxygen fluxes.

The Boehm titration data for the catalysts prepared by different modification gas flows are shown in Table 5, and the FTIR spectra are shown in Figure 8.

As can be seen from Table 5, the effect of oxygen modification flow on the functional groups of the catalyst surface is similar to that of the oxygen modification time. The acidic and basic functional groups on the catalyst surface cannot co-exist in large numbers, and there is a competing relationship between them.

As can be seen from Figure 8, a different oxygen modification gas flow has a significant influence on the number of the functional groups. The functional groups have changed significantly at 2360~2332 and 1100. When the oxygen modification gas flow was small, the high-energy oxygen particles ionized from the plasma were fewer, and could be taken away by the air. Thus, there were not enough high-energy particles to modify the catalyst, and its functional groups did not change significantly. When the oxygen modification gas flow was large, the plasma ionized more high-energy oxygen particles; the particles interacted with the functional groups on the catalyst surface and oxidized the unsaturated functional groups C≡C and C≡N to C=O and N=O, respectively, in a short time. However, when the time was too long, the high energy oxygen particles would oxidize the unsaturated functional groups completely, which meant there were no unsaturated functional groups on the catalyst surface, so that the reaction of nitrous into nitro and carbonyl into carbon dioxide disappeared in the denitration experiment [22,23]. Combined with Table 5 and Figure 8, it can be seen that the number of basic functional groups on the catalyst surface is the greatest and the denitration effect is the best when the oxygen flow is 40 mL/min, indicating that the catalyst surface contains more active sites under those conditions.

## 4. Conclusions

In this paper, fly ash and bentonite were mixed in a certain proportion and used as raw material to prepare the denitration catalyst. The effects of different modification times, modification power levels, and modification gas flow rates on denitration efficiency of the catalyst were studied. We can draw the following conclusions:

(1) The catalyst can be prepared by plasma modification technology, and the plasma power, time and gas flow of the modification have an effect on the denitration efficiency of the catalyst. The optimum plasma power is 60 W, the optimum modification time is 20 min, and the optimum gas flow rate is 40 mL/min.
(2) The catalyst was modified by the plasma; the denitration effect of the catalyst modified by the plasma was significantly higher than that of the unmodified catalyst.
(3) The different preparation conditions can make different functional groups and alter the efficiency of denitration. The effect of basic functional groups N=O on denitration was significant, followed by C=O. The effect of phenolic hydroxyl was not obvious, and may be synergistic with other functional groups.

## Figures and Tables

**Table 1:** Elemental analysis of fly ash.

Metal Type	Sample Mass	Nitrogen Content/%	Carbon Content/%	Hydrogen Content/%	C/N
Fly ash	3.726	0.04	2.191	0.14	54.37
bentonite	3.769	0.016	0.174	1.678	10.93

**Table 2:** ICP analysis of fly ash.

Metal Type	>5 ppm	1~5 ppm	0.5~1 ppm	0.1~0.5 ppm	<0.1 ppm	Metal Number
Fly ash	K Mg	Ba Mn Sr	Cr	Cu Nb Ni Ru U V Zn	Be Cd and other 19 kinds	32
bentonite	K Mg	none	Ba Sr Zn	Ce Cr Cu Mn	Pb Ru Be Dy and other 20 kinds	31

**Table 3:** Boehm titration for the catalyst with different modification times (unit: mmol/g).

Sample	Acid Functional Groups	Basic Functional Groups	Phenolic Hydroxyl Groups	Carboxyl
10 min	3.6000	0.0000	0.0000	0.0172
20 min	0.4500	2.8000	0.0035	0.0033
30 min	3.6500	0.0500	0.0082	0.0075
40 min	3.8000	0.0000	0.0107	0.0078

**Table 4:** Boehm titration with different modified power levels (unit: Mmol/g).

Sample	Acid Functional Groups	Basic Functional Groups	Phenolic Hydroxyl Groups	Carboxyl
30 W	0.1700	2.3800	0.0054	0.0076
60 W	0.4500	2.8000	0.0035	0.0033
90 W	0.1000	2.4500	0.0000	0.0170

**Table 5:** Boehm titration for different modification gas flows (Unit: mmol/g).

Sample	Acid Functional Groups	Basic Functional Groups	Phenolic Hydroxyl Groups	Carboxyl
20 mL/min	4.3000	0.0000	0.0055	0.0072
40 mL/min	0.4500	2.8000	0.0035	0.0033
60 mL/min	4.4500	0.0000	0.0100	0.0087
